# Emotional and behavioral outcomes and quality of life in school-age children born as late preterm: retrospective cohort study

**DOI:** 10.3325/cmj.2017.58.332

**Published:** 2017-10

**Authors:** Branka Polić, Andreja Bubić, Julije Meštrović, Joško Markić, Tanja Kovačević, Ivanka Antončić Furlan, Ivan Utrobičić, Ivana Kolčić

**Affiliations:** 1 Department of Pediatrics, PICU, University Hospital Center Split, Split, Croatia; 2 Chair for Psychology, Faculty of Social Sciences and Humanities, University of Split, Split, Croatia; 3 Department of Neonatology, University Hospital Center Split, Split, Croatia; 4 Department of Plastic Surgery, University Hospital Center Split, Split, Croatia; 5 Department of Public Health, School of Medicine, University of Split, Split, Croatia

## Abstract

**Aim:**

To determine the effect of late preterm birth and treatment at the intensive care unit (ICU) on school-age children’s emotional and behavioral problems and quality of life (QoL).

**Methods:**

Emotional and behavioral problems and QoL were investigated in 6-12-year-olds who were born late preterm at the University Hospital Center Split in the period from January 2002 to March 2008. The study included 126 late preterm children treated in ICU (LP-ICU group), 127 late preterm children not treated in ICU (LP-non-ICU group), and 131 full-term children treated in ICU (FT-ICU group). Emotional and behavioral difficulties were assessed using the Child Behavior Checklist. QoL was evaluated with the Royal Alexandra Hospital for Children Measure of Function questionnaire. The data was collected via telephone interview with mothers during 2014.

**Results:**

Late preterm children had a nearly 5-fold risk for internalizing problems in comparison with FT-ICU children (OR 4.76, 95% confidence interval [CI] 2.37-9.56 and OR 4.82, 95% CI 2.25-10.37 in LP-ICU and LP-non-ICU children, respectively). They also had a greater risk for externalizing problems (OR 3.08, 95% CI 1.44-6.61 and OR 2.68, 95% CI 1.14-6.28, respectively) and total problems (OR 6.29, 95% CI 2.86-13.83 and OR 7.38, 95% CI 3.08-17.69, respectively) and a considerably increased risk for lower QoL (OR 12.79, 95% CI 5.56-29.41 and OR 5.05, 95% CI 2.04-12.48, respectively).

**Conclusion:**

Children born late preterm had a greater risk for emotional and behavioral problems and lower QoL during childhood than their full-term born peers and they experienced serious health problems upon birth.

Late preterm infants, born between 34^0/7^ and 36^6/7^ weeks of gestational age (GA), have an increased risk of mortality and morbidity in comparison to full-term infants (1). In addition to short-term medical morbidity, late preterm infants are also at a greater risk for long-term neurodevelopmental difficulties and may require special educational support to offset the increased risk of poorer academic performance (2). The limited evidence on long-term cognitive and behavioral functioning of children born late preterm indicates that behavioral problems are more frequent in late preterm born children than in full-term born children (3-5).

Due to recent development in intensive care procedures, the survival rates of premature infants have increased. However, the possibility of health-related issues has also increased, as well as behavioral and learning problems later in the lives of these children (6). Therefore, monitoring various outcomes after treatment in an intensive care unit (ICU) during early neonatal period, including emotional and behavioral problems and quality of life (QoL), has become necessary. Additionally, the measurement of QoL in children has become a mandatory component in clinical research because it is useful for understanding the impact of both disease and medical treatment on the long-term outcomes (7).

Extremely preterm-born children, who were more frequently investigated regarding their long-term outcomes, have a greater risk for emotional and behavioral problems, more problems related to executive functions and learning skills, and reduced QoL during childhood (6,8). Attention and learning problems of prematurely born children are associated with educational disadvantages and have a negative effect on QoL at school age (9).

The majority of previous studies were based on the comparison of very or moderately preterm-born children and healthy full-term children (10,11). Only a few studies analyzed the outcomes in healthy late preterm born infants who were not admitted to the ICU as a comparison group for late preterm infants who were admitted to the ICU (12,13). Unfortunately, most authors did not report the influence of prenatal and neonatal complications and treatment methods used in the ICU on the children’s outcomes.

This study was designed to clarify the association of late preterm birth and ICU treatment and children’s emotional and behavioral problems and QoL at school age (6 to 12 years). The first aim was to assess the emotional and behavioral problems and QoL of school-age children born late preterm who were treated in the ICU and to compare these outcomes with the outcomes of children who were also born late preterm but were not admitted to the ICU and full-term infants who were admitted to the ICU. The second aim was to identify the risk factors that might predict internalizing, externalizing and total emotional and behavioral problems and poorer QoL in school-age children.

## PARTICIPANTS AND METHODS

### Participants

The study population consisted of school children aged between 6 and 12 years who were born at the Department of Obstetrics and Gynecology of the University Hospital Centre Split from January 2002 to March 2008. Late preterm birth was defined as birth between 34^0/7^ and 36^6/7^ weeks of GA, while full-term infants were born between 37^0/7^ and 40 weeks of GA. GA was determined according to the last menstrual period and confirmed with ultrasound for all the children. Exclusion criteria in all three groups of children included metabolic and other genetic disorders, congenital malformations or any syndrome (severe conditions that cause significant emotional and behavioral disturbances later in life), multiple pregnancies, mother’s drug addiction, and the failure to provide informed consent for this study.

The indications for treatment in the ICU, defined by the British Association of Perinatal Medicine, included complex problems requiring 24-hour medical care, the need for respiratory support via tracheal tube or nasal continuous positive airway pressure (NCPAP), <1000 g weight receiving NCPAP, the need for major emergency surgery with preoperative and postoperative care longer than 24 hours, the need for complex clinical procedures, and any other instability of infant (14). Primary diagnoses for ICU treatment were categorized into broad groups of hypoxia, infections, respiratory distress syndrome, and others (pneumonia, hypoglycemia, convulsions, meconium aspiration syndrome, gastrointestinal diseases, and so on). Late preterm infants who did not receive the ICU treatment were those who did not need medical care for more than three days. The medical care included, for example, phototherapy, temperature control, infusion or only continuous observation.

Inclusion criteria were met by 136 late preterm infants who received the ICU treatment (LP-ICU) and were included in the study. Given that there were more late preterm infants who were not admitted to the ICU (LP-non-ICU) and full-term infants who received treatment in the ICU (FT-ICU), we used the random number generator option in the Excel to select 136 children both of these groups from the list of children. Since the maternal response was 92.6% in the LP-ICU group, 93.4% in the LP-non-ICU, and 96.3% in the FT-ICU, the study groups included 126, 127, and 131 children, respectively.

### Procedures

Medical records served as a source of information on complications during pregnancy (preeclampsia, premature rupture of membranes, chorioamnionitis, intrauterine growth retardation, and so on), GA, mode of delivery (vaginal or cesarean section), Apgar scores, birth weight, diagnoses for the admission to the ICU, need for mechanical ventilation and the number of days a child was treated in the ICU. Mother’s telephone contacts were also retrieved from the medical records.

Emotional and behavioral difficulties and competencies were assessed using the parent Child Behavior Checklist (CBCL) for ages 6-18 years (15). The CBCL consists of 113 problem items (questions) in nine scales, ie, eight syndrome scales and one for other problems. The syndrome scales include anxious/depressed, withdrawn/depressed, somatic complaints, social problems, thought problems, attention problems, rule-breaking behavior, and aggressive behavior. The first three syndrome scales determine internalizing problems (internalizing broadband scale), and the last two syndrome scales combined determine externalizing problems (externalizing broadband scale). Internalizing problems refer to the problems that are mainly within oneself. Externalizing problems refer to problems involving conflicts with other people and their expectations for the child. High scores reflect numerous problems that indicate clinically important deviance and the need for professional intervention. The Croatian version of the CBCL questionnaire had been previously validated in a sample of 3309 healthy children from the general population in Croatia (16). Each question is rated as not true (0), somewhat and or sometimes true (1 point), or very true or often true (2 points). The sum of the raw scores on both internalizing and externalizing broadband scales and for total emotional and behavioral problems was divided according to the CBCL manual (15) into two categories: below clinical range (normal and borderline range) and clinical range (>90th percentile, which represents the cut-off for clinical range of problems). The actual cut-off scores were taken from the study based on a representative sample of healthy Croatian children (16). Clinical outcome was defined as a raw score greater than 12 points for both genders on the internalizing scale, >13 and >16 points on the externalizing scale, and >37 and >44 on total problems for girls and boys, respectively, representing cut-off points for the >90th percentile for normative distribution among children in Croatia (16).

QoL was evaluated using the Royal Alexandra Hospital for Children Measure of Function (RAHC MOF) questionnaire, generic QoL scoring system (17). The instrument consists of 10 categories, each containing 10 scales. The clinical rating scales are comprised of targeted questions that take into account the strength and impact of symptoms as follows: physical disability and limitation of movements, emotional and behavioral disturbance, deviation from normal growth and development and limitations and disturbances in social life, the quality and extent of the relationship with family members and friends, school performance, and extracurricular activities. The RAHC MOF questionnaire is scored from 1 to 100. A score from 1 to 30 indicates a high level of health problems and consequent poor QoL. These children are significantly impaired in several areas of development and physical and cognitive functioning and health. A child with a score from 31 to 70 on the RAHC MOF questionnaire shows a fair QoL with possible developmental delay in one or more areas. A score from 71 to 100 reflects minimal health problems and good QoL (17). The Croatian version of this questionnaire was previously validated on the population of children who were treated in ICU (18,19).

After the final sample and exposure groups were defined, three well-trained interviewers (MJ, RT, and HS) collected the data via telephone interview with mothers during 2014. Telephone numbers were given randomly to the interviewers and no other data were revealed. Each interviewer was instructed first to ask the questions from the CBCL and RAHC MOF questionnaire and then to collect information from the mothers on their education level (primary, high school, or university) and the socioeconomic status of the family. Socioeconomic status of the family was determined on the basis of the education and occupation of both parents and family income, and it was categorized as poor, average, or good. Poor socioeconomic status meant that parents finished primary school and/or had jobs that did not require any qualifications or did not have a job and/or that family income was lower than the Croatian average. Average socioeconomic status included high school degree and/or jobs that matched education level and average Croatian income. Good socioeconomic status meant that parents had a university degree and/or jobs matching their education and/or income higher than the Croatian average.

### Statistical analysis

Categorical data were shown as numbers and percentages, and numerical data were presented as medians with interquartile ranges due to the non-normal distribution of the data (tested by Kolmogorov-Smirnov test). Differences between LP-ICU children, LP-non-ICU children, and FT-ICU children with respect to several background demographic characteristics and medically relevant variables were tested using the χ^2^ test for categorical data and non-parametric Kruskal-Wallis test for continuous data. The extent of emotional and behavioral problems expressed as raw CBCL scores on the internalizing, externalizing, and total problem scales in three subsamples were compared using the Kruskal-Wallis test (comparison for three groups) and Mann-Whitney test was used in the post-hoc analysis for the comparison of two groups.

Finally, multivariate logistic regression was used to identify the factors that might be used for predicting clinical outcome for the internalizing, externalizing, and total emotional and behavioral problems and QoL. All four logistic regression models included the following predictor variables: gender (girls as a reference group), child’s age when tested, mother’s age at delivery, complications during pregnancy, mode of delivery (vaginal delivery as a reference group), Apgar score at 5 minutes, days of treatment in the ICU, mechanical ventilation, mother’s educational attainment (higher education as a reference group), family socioeconomic status (good status as a reference group), and study group (FT-ICU as a reference group).

The data were analyzed using SPSS 20.0 (IBM SPSS Statistics for Windows, v. 20.0, released 2011. IBM Corp., Armonk, NY, USA). *P* < 0.05 was considered statistically significant.

## RESULTS

The three study groups did not differ in gender composition, children’s age at time of data collection or socioeconomic status and mothers’ educational level. However, there were statistically significant differences between the study groups in the prevalence of complicated pregnancies, mode of delivery, Apgar score, and medically relevant criteria associated with prematurity, such as GA and birth weight (Table 1). Children in the LP-ICU and FT-ICU groups did not differ significantly according to the primary diagnosis at birth. However, a greater proportion of children in the LP-ICU group required mechanical ventilation and received longer treatment in the ICU.

**Table 1:** Characteristics of school-aged children born late preterm or full term*

	No. (%) of subjects			
	LP-ICU (n = 126)	LP-non-ICU (n = 127)	FT-ICU (n = 131)	*P*
Gender				
male	71 (56.3)	73 (57.5)	78 (59.5)	0.871
female	55 (43.7)	54 (42.5)	53 (40.5)	
Child’s age when tested (years)				
≤9	46 (36.5)	47 (37.0)	47 (35.9)	0.982
>9-12	80 (63.5)	80 (63.0)	84 (64.1)	
Gestational age (weeks)				
34^0/7^-34^6/7^	36 (28.6)	18 (14.2)	0	<0.001
35^0/7^-36^6/^7	90 (71.4)	109 (85.8)	0	
≥37	0	0	131 (100)	
Complicated pregnancy				
no	45 (35.7)	61 (48.0)	83 (63.4)	<0.001
yes	81 (64.3)	66 (52.0)	48 (36.6)	
Mode of delivery				
vaginal	78 (61.9)	103 (81.1)	98 (75.4)	0.002
Cesarean section	48 (38.1)	24 (18.9)	32 (24.6)	
Birth weight (g; median, IQR)	2600 (613)	2600 (500)	3700 (650)	<0.001
Apgar score 5 minutes (median, IQR)	8 (2)	9 (2)	9 (3)	<0.001
Primary diagnosis at birth				
healthy	0	112 (88.2)	0	0.509^†^
hypoxia	45 (35.7)	0	36 (27.5)	
infection	34 (27.0)	0	36 (27.5)	
RDS	24 (19.0)	0	30 (22.9)	
other	23 (18.3)	15 (11.8)	29 (22.1)	
Mechanical ventilation				
no	89 (70.6)	127 (100)	114 (87.0)	0.002^†^
yes	37 (29.4)	na	17 (13.0)	
Duration of ICU treatment (days; median, IQR)	8.0 (4.0)	na	5.0 (3.0)	<0.001^†^
Family socio-economic status				
poor	9 (7.2)	14 (11.1)	11 (8.4)	0.424
average	88 (69.8)	85 (67.5)	81 (61.8)	
good	29 (23.0)	27 (21.4)	39 (29.8)	
Mothers’ age at delivery (years)				
<25	27 (21.4)	31 (24.4)	22 (16.8)	0.315
25-29	40 (31.8)	40 (31.5)	55 (42.0)	
30-34	35 (27.8)	40 (31.5)	37 (28.2)	
≥35	24 (19.0)	16 (12.6)	17 (13.0)	
Order of birth				
firstborn	76 (60.3)	68 (53.5)	71 (54.2)	0.050
second-born	26 (20.6)	45 (35.4)	34 (26.0)	
between third- and fifth-born	24 (19.0)	14 (11.0)	26 (19.8)	
Mothers’ educational level				
primary or high school	86 (68.3)	86 (67.7)	94 (71.8)	0.746
university	40 (31.7)	41 (32.3)	37 (28.2)	

*Abbreviations: LP-ICU – late preterm infant treated in the intensive care unit, LP-non-ICU – late preterm infant not treated in ICU, FT-ICU – full-term infant treated in ICU, IQR – interquartile range, RDS – respiratory distress syndrome, na – not applicable.

†LP-ICU vs FT-ICU group.

LP-ICU children had the highest average scores on both CBCL broadband scales and total behavioral problems, followed by the LP-non-ICU group. This difference was significant for all between-group comparisons, except for externalizing behavioral problems in LP-ICU and LP-non-ICU groups (Table 2). The average total CBCL score in the LP-ICU group was twice as high as in the FT-ICU group. There was no difference between girls and boys in the average scores in behavioral problems, but the boys in the LP-ICU and LP-non-ICU groups had significantly worse QoL in comparison with girls.

**Table 2:** Raw scores in Child Behavior Checklist (CBCL) internalizing broadband scale, externalizing broadband scale, and total score and quality of life (QoL) in children born late preterm and full term*

	Score (median, IQR)^†^											
	LP-ICU				LP-non-ICU				FT-ICU			
	total (n = 126)	girls (n = 55)	boys (n = 71)	*P^‡^*	total (n = 127)	girls (n = 54)	boys (n = 73)	*P*^§^	total (n = 131)	girls (n = 53)	boys (n = 78)	*P*^II^
CBCL												
internalizing	11.5 (12.3)	12.0 (10.0)	11.0 (13.0)	0.007	8.0 (12.0)	8.5 (8.0)	8.0 (14.0)	<0.001	4.0 (6.0)	4.0 (6.75)	5.0 (7.0)	<0.001
*P*^¶^		0.992				0.899				0.120		
externalizing	10.0 (10.3)	8.0 (10.0)	10.0 (12.0)	0.112	8.0 (8.0)	8.0 (8.0)	8.0 (9.0)	<0.001	5.0 (8.0)	4.0 (4.75)	6.0 (9.0)	<0.001
*P*^¶^		0.086				0.604				0.256		
total	37.0 (33.0)	37.0 (35.0)	47.0 (36.0)	0.009	29.0 (21.0)	33.0 (23.0)	35.0 (32.0)	<0.001	15.0 (21.0)	16.0 (15.0)	20.5 (25.0)	<0.001
*P*^¶^		0.051				0.455				0.099		
QoL												
	72.0 (14.0)	75.0 (14.0)	68.0 (14.0)	<0.001	75.0 (10.0)	78.0 (11.0)	74.0 (14.0)	<0.001	85.0 (11.0)	86.0 (14.0)	85.0 (11.0)	<0.001
*P*^¶^		0.028				0.028				0.502		

*Abbreviations: IQR – interquartile range, LP-ICU – late preterm infant treated in the ICU, LP-non-ICU – late preterm infant not treated in the ICU, FT-ICU – full-term infant treated in the ICU.

†Comparison of totals for all three study groups, *P* < 0.001 for all.

‡Post-hoc test, comparison of total scores in LP-ICU vs LP-non-ICU.

§Post-hoc test, comparison of total scores in LP-non-ICU vs FT-ICU.

IIPost-hoc test, comparison of total scores in LP-ICU vs FT-ICU.

¶Gender comparison.

The highest incidence of the total clinically relevant emotional and behavioral problems was found among boys in the LP-ICU group, with 43.7% of the boys being classified as having the clinical range of problems, followed by 38.2% girls from the same group (Table 3). In contrast, in the FT-ICU group, only 7.7% of the boys and 7.5% of the girls were classified as having clinical emotional and behavioral problems. Differences between LP-ICU and FT-ICU groups were statistically significant in all domains of behavioral problems and QoL (*P* < 0.001 for all), while LP-ICU and LP-non-ICU groups differed in the incidence of clinically relevant externalizing and total problems and QoL.

**Table 3:** Cumulative incidence of clinical outcomes for internalizing, externalizing and total behavioral problems, using cut-off values for raw scores >90th percentile on the Child Behavior Checklist (CBCL) obtained from the reference healthy population of children in Croatia (16) and quality of life (QoL)*

	No. (%) of children^†^											
	LP-ICU				LP-non-ICU				FT-ICU			
	total (n = 126)	girls (n = 55)	boys (n = 71)	*P^‡^*	total (n = 127)	girls (n = 54)	boys (n = 73)	*P*^§^	total (n = 131)	girls (n = 53)	boys (n = 78)	*P*^II^
CBCL												
internalizing	53 (42.1)	23 (41.8)	30 (42.3)	0.081	40 (31.5)	14 (25.9)	26 (35.6)	<0.001	15 (11.4)	3 (5.7)	12 (15.4)	<0.001
*P*^¶^		0.961				0.245				0.086		
externalizing	37 (29.4)	16 (29.1)	21 (29.6)	0.015	21 (16.5)	8 (14.8)	13 (17.8)	0.076	12 (9.2)	5 (9.4)	7 (9.0)	<0.001
*P*^¶^		0.953				0.653				0.929		
total	52 (41.3)	21 (38.2)	31 (43.7)	0.022	35 (27.6)	15 (27.8)	20 (27.4)	<0.001	10 (7.6)	4 (7.5)	6 (7.7)	<0.001
*P*^¶^		0.535				0.962				0.976		
QoL												
fair	60 (47.6)	19 (34.5)	41 (57.7)	<0.001	30 (23.6)	8 (14.8)	22 (30.1)	<0.001	9 (6.9)	4 (7.5)	5 (6.4)	<0.001
good	66 (52.4)	36 (65.5)	30 (42.3)		97 (76.4)	46 (85.2)	51 (69.9)		122 (93.1)	49 (92.5)	73 (93.6)	
*P*^¶^		0.010				0.044				0.801		

*Abbreviations: LP-ICU – late preterm infant treated in the ICU, LP-non-ICU – late preterm infant not treated in the ICU, FT-ICU – full-term infant treated in the ICU.

†Comparison of totals for all three study groups, *P* < 0.001 for all.

‡Post-hoc test, comparison of total scores in LP-ICU vs LP-non-ICU.

§Post-hoc test, comparison of total scores in LP-non-ICU vs FT-ICU.

IIPost-hoc test, comparison of total scores in LP-ICU vs FT-ICU.

¶Gender comparison.

The multivariate logistic regression models identified several variables associated with the clinically relevant emotional and behavioral problems and fair QoL (Table 4). The most pronounced risk factor for both broadband scales of behavioral problems, total behavioral problems, and fair QoL was late preterm birth in comparison with full-term birth. Both groups of children born late preterm had 4.8 times greater risk for problems in the clinical range in the internalizing domain, more than 2.5-fold risk for clinically relevant externalizing problems, and more than 6-fold risk for total clinical behavioral problems in comparison with the FT-ICU children. Children born late preterm also had a substantially greater risk for fair, rather than good, QoL in comparison with the full-term born children. Among other risk factors, a longer treatment in the ICU was associated with externalizing problems and total behavioral problems, and mechanical ventilation was associated with worse QoL.

**Table 4:** Multivariate logistic regression analysis of risk factors for the clinical outcome in the internalizing, externalizing, and total behavior problems measured with Child Behavior Checklist (CBCL) and fair quality of life (QoL)*

		OR (95% CI)		
	CBCL internalizing	CBCL externalizing	CBCL total	Fair QoL
Male gender (reference group: girls)	1.34 (0.81-2.21)	1.00 (0.57-1.76)	1.00 (0.59-1.69)	2.16 (1.25-3.74)
Child’s age when tested (years)	0.86 (0.74-0.99)	0.80 (0.67-0.94)	0.85 (0.72-0.99)	1.14 (0.97-1.33)
Mother’s age at delivery (years)	0.93 (0.88-0.97)	0.95 (0.90-1.01)	0.94 (0.89-0.99)	1.02 (0.97-1.07)
Complicated pregnancy	0.94 (0.56-1.59)	1.02 (0.57-1.86)	1.02 (0.58-1.78)	1.34 (0.75-2.39)
Cesarean section delivery (reference group: vaginal delivery)	0.75 (0.41-1.37)	0.83 (0.42-1.65)	0.62 (0.32-1.19)	0.89 (0.47-1.71)
Apgar score at 5 minutes	0.99 (0.84-1.19)	1.09 (0.89-1.34)	1.12 (0.93-1.38)	0.97 (0.80-1.18)
Duration of ICU treatment (days)	1.05 (1.00-1.10)	1.06 (1.01-1.11)	1.08 (1.03-1.14)	0.99 (0.94-1.04)
Mechanical ventilation	1.34 (0.59-3.03)	1.48 (0.62-3.53)	2.05 (0.86-4.89)	4.03 (1.69-9.60)
Mothers’ educational level (higher education is reference group)	0.63 (0.35-1.15)	0.95 (0.47-1.90)	0.65 (0.34-1.25)	1.11 (0.58-2.14)
Family socioeconomic status				
good	reference	reference	reference	reference
medium	1.84 (0.93-3.61)	1.59 (0.73-3.48)	2.44 (1.14-5.24)	0.88 (0.43-1.80)
poor	1.35 (0.45-4.01)	1.87 (0.57-6.12)	4.22 (1.39-12.84)	2.57 (0.89-7.43)
Study group				
FT-ICU	reference	reference	reference	reference
LP-ICU	4.76 (2.37-9.56)	3.08 (1.44-6.61)	6.29 (2.86-13.83)	12.79 (5.56-29.41)
LP-non-ICU	4.82 (2.25-10.37)	2.68 (1.14-6.28)	7.38 (3.08-17.69)	5.05 (2.04-12.48)

*Abbreviations: OR – odds ratio, CI – confidence interval, ICU – intensive care unit, FT-ICU – full-term infant treated in the ICU, LP-ICU – late preterm infant treated in the ICU, LP-non-ICU – late preterm infant not treated in the ICU.

## DISCUSSION

We found that children born late preterm experienced emotional and behavioral problems more frequently and more intensively than full-term born children at school age and that they had poorer QoL. This is in accordance with the previous findings (3-5). However, our results add some new insights, since this is the first study in which the outcomes in late preterm children have been compared with the outcomes in full-term children who were not healthy upon birth, but suffered serious health conditions that required ICU treatment. This particular study design revealed that even children born late preterm who were healthy upon birth demonstrated more frequent clinically relevant internalizing and total behavioral problems and poorer QoL in comparison with full-term children who were treated in the ICU. On the other hand, children born late preterm who were treated in the ICU just after birth had worse outcomes in the clinical range of externalizing and total behavioral problems, and poorer QoL at school age in comparison with healthy children born late preterm. A strikingly high incidence rate of serious, clinically relevant total behavioral problems was recorded among boys (44%) and girls (38%) born late preterm treated in the ICU in comparison with 8% in both genders in the FT-ICU group. These rates are among the highest ones found (4,20-22) and could be compared to the findings by Saigal et al (11), who reported that as many as 40% of extremely immature infants, at the age of eight, had emotional and behavioral difficulties in contrast to 17% of full-term children. A similar study among 4-year-olds in the Netherlands revealed that only 8% of moderately preterm children (9% among boys and 6% among girls) and 5% of term-born children had clinically relevant total behavioral problems (10).

One of the possible explanations why LP-ICU participants had high CBCL scores, and consequently high rates of behavioral problems, may partly be related to the fact that they received longer treatment in the ICU while FT-ICU infants received shorter ICU treatment. Prolonged stay in the ICU of LP-ICU infants is a result of interaction between immaturity and underlying health impairments. Another possible explanation for high rates of behavioral problems among children born late preterm found in this study could be the failure of health care and schooling systems in recognizing these conditions until later school age. This could have led to learning difficulties (3), which could have exacerbated further problems in the development and behavior of these children. Regarding the FT-ICU group, we found that they did not differ from normative population sample of Croatian children in total behavioral problems (16).

Higher mortality and morbidity in premature infants are primarily associated with immaturity, especially with immature brain and lungs. Early interruption of maturation processes of the brain has been suggested as the main reason for negative long-term outcome (23,24). Additionally, treatment procedures in the ICU, stressful environment and acute painful events may lead to the long-term behavior disturbances (25). Unfortunately, it is difficult to differentiate between the influences of immaturity, complications of immaturity, and diseases, which require the ICU admission and the influence of the treatment in the ICU on the behavioral outcomes during childhood. We tried to achieve this by including two control groups, ie, late preterm children who were not admitted to the ICU immediately after birth and full-term infants admitted to the ICU. Our results imply that children born late preterm who were admitted to the ICU had more difficulties in externalizing and total behavioral problems than late preterm children not admitted to the ICU. One previous study showed no difference in the outcomes between such two groups (13), while in another study, the behavioral problems were more frequent in 3-year-olds who were born late preterm and treated in the ICU than children born late preterm who were not treated in the ICU (12). Also, we showed that the children born late preterm who were not admitted to the ICU because they did not have serious health problems had worse scores on both internalizing and externalizing subscales and total problems in comparison with full-term children who were admitted to the ICU. This means that even those children who were healthy, but prematurely born, exhibit worse behavioral problems during childhood than full-term children who had health conditions upon birth serious enough to be admitted to the ICU.

Furthermore, the retrospective cohort study design enabled us to identify some risk factors that were associated with the clinically relevant behavioral problems. The most prominent risk factor for behavioral problems was late preterm birth in comparison with full-term birth. The other risk factor was the length of treatment in the ICU. The number of days the child was treated in the ICU is basically a proxy for the seriousness of the health condition that required admittance to the ICU. We found that a longer treatment in the ICU was associated with externalizing problems and total behavioral problems. As the complications of immaturity are the reason for longer stay in the ICU, we still cannot ignore the impact of the treatment in the ICU on the behavior of children born late preterm. A possible reason could be that a certain percentage of deliveries are unnecessarily completed by elective Cesarean section at earlier gestational age (26,27), which is significantly associated with longer treatment in the ICU (3). Therefore, all efforts must be directed toward prolonging the pregnancy with specific obstetric interventions (eg, tocolysis) to reduce the number of premature births. In addition, further progress in intensive care treatment will reduce possible negative consequences of a prolonged stay in the ICU.

Our study indicated that children born late preterm generally had worse QoL in comparison with full-term children, which is in line with the results of the previous studies (8,9,21,28). We also investigated the impact of some neonatal and socio-demographic factors on the QoL in children. Logistic regression analysis showed that the children who received mechanical ventilation had greater probability for worse QoL at school age. Mechanical ventilation is a significant risk factor for cognitive problems in prematurely born children, and as these children later have poorer school performance than full-term children (29), this may cumulatively lead to the QoL deterioration. Additionally, our results confirmed that boys had higher risk for worse QoL than girls, which was also found among extremely preterm children (9). Males are more vulnerable and have higher mortality and morbidity rates, especially those born prematurely. Boys also need a longer period of oxygen support (9). However, the question is whether the reason for such a correlation is male gender or other confounding factors.

The potential limitation of the study is a relatively small sample size and data collection carried out by telephone interview with mothers. Parents may provide different results than their children in the assessment of QoL (30). However, we do not think that this had an important influence on the conclusions of this study due to the fact that in all three study groups the mothers provided the answers. A slightly different result for between-group comparisons of average raw scores and comparison of incidence rates of clinically relevant behavioral problems requires additional explanation. All raw scores comparisons were significantly different between the groups, but some differences became insignificant when the incidence rates were used. The reason are different cut-off values for boys and girls for externalizing broadband scale and for total behavioral problems scale (16).

The strengths of this study include retrospective cohort study design, high response rate, and the analysis of the influence of the prenatal, neonatal, and socioeconomic factors on the observed outcomes. Lastly, but most importantly, this study compared the emotional and behavioral outcomes in late preterm infants who were admitted to the ICU and the outcomes of late preterm infants who were not admitted to the ICU. This allowed us to make conclusions about the influence of both prematurity and prematurity burdened with health impairments. Also, this is the first study to our knowledge where the control group was the group of full-term children with serious health conditions upon birth. Fewer emotional and behavioral problems in this group of children suggested that serious health conditions and ICU treatment had a stronger impact on children born late preterm than on children born full term. These results should encourage further evaluation of the interaction between prematurity, health problems upon birth, and the ICU treatment and their influence on long-term behavioral outcomes and QoL in children born late preterm. This is particularly important because immaturity and related problems are associated with higher level of stress in mothers of children born late preterm, which can last a long time after birth (31).

In conclusion, this study has found that children born late preterm had a greater risk of emotional and behavioral problems during school-age and worse QoL than full-term children. Also, late preterm children admitted to the ICU had more emotional and behavioral problems and lower QoL than children born late preterm who were not admitted to the ICU. All of these findings suggest that health problems at birth and treatment in the ICU have a negative impact and can amplify long-term consequences on the behavior and QoL of children born late preterm. The direct policy implication is the need for increased medical and psychosocial supervision of late preterm children during their preschool age to perceive the early signs of emotional and behavioral problems. The early detection of problems and proper intervention could diminish long-term consequences and improve QoL of children born late preterm at school-age and later in life.
